# Fabrication of Li/In Double-Sided Diffusion Contacts in Planar High-Purity Germanium Detectors and Their Low-Temperature X-Ray Response

**DOI:** 10.3390/ma19143143

**Published:** 2026-07-22

**Authors:** Meng Cao, Zexin Wang, Yanggang Jia, Qingzhi Hu, Zhaoran Guan, Haofei Huang, Linjun Wang, Jian Huang

**Affiliations:** 1 State Key Laboratory of Materials for Advanced Nuclear Energy & School of Materials Science and Engineering, Shanghai University, Shanghai 200444, China; wangzexin0208@shu.edu.cn (Z.W.); ygjia1999@163.com (Y.J.);; 2 Zhejiang Institute of Advanced Materials, Shanghai University, Jiashan 314113, China; 3 Shanghai Engineering Research Center for Integrated Circuits and Advanced Display Materials, Shanghai University, Shanghai 200444, China

**Keywords:** high-purity germanium detector, diffusion contact, low-temperature dark current, X-ray response

## Abstract

Li n^+^ and In p^+^ diffusion contacts were fabricated on p-type 12N high-purity germanium (HPGe) single crystals by vacuum evaporation of thin-film sources followed by solid-state thermal diffusion. The effects of diffusion temperature on the near-surface structure, morphology, impurity distribution, and device response were systematically investigated. XRD and Raman analyses show that Li diffusion at 100–300 °C and In diffusion at 600–800 °C preserve the bulk Ge crystal structure, whereas higher diffusion temperatures induce surface roughening, near-surface disordering, and interfacial reactions. SIMS depth profiles combined with diffusion simulations confirm effective inward diffusion of both Li and In, with low-concentration tailing that is consistent with defect-assisted diffusion or interfacial trapping. The sample diffused with Li at 200 °C exhibits the lowest dark current, 8.07 × 10^−8^ A at −10 V. The final HPGe device with Li/In diffusion contacts shows a stable synchrotron X-ray photoconductive response, and the net response current increases from 4.48 × 10^−7^ to 1.15 × 10^−6^ A as the incident photon flux increases. These results demonstrate that low-leakage HPGe diffusion contacts require a balance between diffusion-layer formation and near-surface/interface stability, rather than a simple increase in thermal budget.

## 1. Introduction

High-purity germanium (HPGe) detectors combine a high atomic number, a narrow bandgap, excellent carrier transport properties, and exceptionally high energy resolution, making them a key class of semiconductor detectors for γ-ray spectroscopy, nuclear-radiation measurements, low-background physics experiments, and low-energy X-ray detection [1,2]. Recent work on point-contact, segmented, and cryogenic HPGe devices has further emphasized that detector performance is strongly affected by contact design, surface condition, and charge-collection behavior near the electrode region [3,4]. For planar or point-contact HPGe devices, the bulk crystal purity, compensation level, and deep-level defects determine the intrinsic charge-collection capability. In contrast, metal/semiconductor contacts, diffusion layers, and near-surface interfacial states control the low-temperature leakage current, depletion behavior, dead-layer thickness, local electric-field distribution, and low-energy response [5,6]. Dead-layer studies and Monte Carlo efficiency calibrations also show that even small changes in inactive or transition layers can alter the apparent detector efficiency and spectral response [7,8]. Therefore, contact-layer fabrication is not merely a final device-processing step, but a critical link connecting material quality, interfacial stability, and detector performance.

In p-type HPGe devices, Li diffusion is one of the classical approaches for forming n^+^ contact layers. Li has high diffusivity in Ge and can act as a shallow donor, forming a relatively thick n-type diffusion layer that provides electron-injection blocking capability and mechanical robustness [1,6]. However, thick Li-diffused contacts are usually accompanied by a full dead layer and a transition layer; energy deposition within these regions can lead to partial charge collection, low-energy-event misidentification, and enhanced continuum background in measured spectra [5,8]. Measurements and simulations of HPGe dead layers have shown that the inactive-layer thickness and its spatial nonuniformity are major sources of uncertainty in efficiency calibration and low-energy response analysis [7,9]. More recent studies of HPGe dead-layer profiling further indicate that contact geometry, encapsulation, and long-term surface evolution can affect the detector response over a wide energy range [10,11,12]. In addition, Li is chemically active, and diffusion, transfer, and cooling can be affected by oxygen, moisture, and residual surface contamination. When the thermal budget is too high, surface reactions, local roughening, and increased near-surface defect density may enhance surface leakage current and non-ideal carrier injection. Thus, Li-diffused contacts should be optimized not only for diffusion depth, but also for surface morphology, chemical state, and electrical stability.

The formation of a p^+^ contact layer must likewise balance doping effectiveness with interfacial integrity. Indium is a typical acceptor impurity in Ge, and dopant diffusion in Ge is closely coupled to intrinsic point defects, activation state, and thermal-treatment conditions [13,14]. Compared with Li, effective In diffusion in Ge generally requires higher thermal activation, which makes p^+^ surface-contact fabrication more susceptible to surface migration, agglomeration, oxidation, and thermal-stress-induced perturbations [15,16]. Studies of dopant profiling and activation in Ge show that impurity diffusion cannot be treated as a purely Fickian broadening process; instead, it can be affected by defect coupling, dopant deactivation, carrier concentration, and non-ideal boundary conditions [17,18]. Surface-treatment studies on high-purity Ge and GeO_2_-based passivation further demonstrate that chemical preparation and oxide/interfacial states are important for maintaining stable Ge surfaces and suppressing electrically active defects [19,20]. Therefore, for In thin-film-source thermal diffusion contacts, an excessively high temperature may increase the diffusion depth but may also damage near-surface morphology and interfacial stability, ultimately offsetting the electrical benefits of p^+^ contact formation. Despite these advances, the coupled process window for Li/In double-sided diffusion contacts prepared by vacuum evaporation followed by solid-state thermal diffusion remains insufficiently clarified, especially with respect to the continuous relationship among structure, morphology, chemical state, depth distribution, and low-temperature device response. Although these contact strategies have advanced HPGe detector fabrication, they still leave an unresolved processing dilemma. Alternative contact schemes, such as amorphous-semiconductor contacts, implanted contacts, and wraparound Li-diffused electrodes, can improve specific aspects of carrier blocking, leakage-current suppression, or device geometry. However, they do not directly resolve the coupled thermal-diffusion problem in Li/In double-sided contacts. In particular, Li diffusion requires sufficient donor-layer formation while avoiding surface oxidation and roughening, whereas In diffusion requires higher thermal activation but may induce stronger surface migration, agglomeration, and interfacial perturbation. Therefore, the key issue is not simply whether Li or In can diffuse into Ge, but how to define a process window that balances diffusion depth, near-surface stability, and low-temperature leakage-current suppression. This relationship remains insufficiently established for Li/In double-sided diffusion contacts prepared from evaporated thin-film sources.

Based on these considerations, p-type 12N HPGe single crystals were used as substrates in this work, and Li n^+^ and In p^+^ contact layers were separately prepared by vacuum evaporation of thin-film sources followed by solid-state thermal diffusion. Li thin films were diffused at 100–400 °C in an inert-atmosphere glovebox, whereas In thin films were diffused at 600–900 °C in a three-zone tube furnace. It should be noted that the actual surface supply in thin-film-source thermal diffusion is affected by both the pre-deposited film thickness and the subsequent thermal treatment process. Therefore, the actual diffusion profile may deviate from the ideal diffusion model, particularly in the low-concentration tail region. Based on this consideration, diffusion simulations were combined with SIMS depth profiling in this work to analyze the evolution trends of the Li/In diffusion fronts and the main diffusion regions. By combining XRD, Raman spectroscopy, SEM/AFM, XPS, synchrotron μ-XRF, SIMS, low-temperature I–V measurements, and synchrotron X-ray response tests, this study establishes the relationship among diffusion temperature, near-surface structural evolution, impurity diffusion depth, and device electrical response. The aim of this work is not to describe thin-film-source thermal diffusion as an entirely new process, but to clarify the competition among diffusion-layer formation, interfacial stability, and defect-induced leakage in Li/In double-sided diffusion contacts, and to identify a reasonable process window and failure boundary for HPGe detector fabrication.

## 2. Materials and Methods

### 2.1. Experimental Materials and Device Structure

P-type HPGe single crystals with a purity of 12N were purchased from Umicore (Brussels, Belgium) and used as the substrates. The sample dimensions were approximately 10 mm × 10 mm × 5 mm. Before diffusion, the sample surfaces were subjected to chemical mechanical polishing and cleaning to remove the damage layer, particulate contaminants, and polishing residues generated during cutting and mechanical processing, thereby providing clean and flat contact surfaces for subsequent thin-film evaporation and thermal diffusion. The device adopted a planar double-sided diffusion contact structure. An n^+^ contact layer was formed on one side by Li diffusion, while a p^+^ surface contact region was formed on the opposite side by In diffusion. After diffusion, Au electrodes were deposited on both sides for low-temperature electrical measurements and X-ray response tests.

### 2.2. Preparation of Li/In Diffusion Contacts

In this work, Li/In double-sided diffusion contacts were prepared using a thin-film-source solid-state thermal diffusion method. The Li source had a purity of 99.95% and was purchased from Dongguan Canrd Laboratory Equipment Technology Co., Ltd. (Dongguan, China). Solid Li foils and solid In pellets were used as the evaporation sources for Li and In deposition, respectively. For each evaporation run, at least approximately 0.25 g of Li foil and 1.0 g of In pellets were loaded into the evaporation source. These loaded masses were intentionally higher than the theoretical deposited masses because part of the evaporated material was inevitably deposited on the chamber wall, substrate holder, shielding components, and other non-sample regions during thermal evaporation. Therefore, the source materials were supplied in excess, while the effective impurity supply on the HPGe surface was controlled by the deposited film thickness. In this method, Li or In thin films with controlled thicknesses were first deposited on the HPGe surface by vacuum evaporation and used as surface impurity sources for subsequent diffusion. Thermal treatment was then applied to drive impurity diffusion from the surface into the near-surface region of Ge. Because Li and In differ significantly in chemical activity, diffusion temperature range, and processing stability, different thermal diffusion equipment and atmosphere-control methods were used for the two elements.

#### 2.2.1. Preparation of Li-Diffused Contacts

Li diffusion was used to form an n^+^ contact layer on one side of the p-type HPGe substrate. First, the pretreated HPGe sample was fixed on the sample stage of an integrated thermal evaporation and glovebox system (VZZ-300S, Beijing MicroNano Vacuum Technology Co., Ltd., Beijing, China), and a 100 nm Li thin film was deposited on the target surface. After deposition, the sample was transferred into a glovebox filled with an inert atmosphere and placed on a hot plate for low-temperature solid-state thermal diffusion. The diffusion temperatures were 100, 200, 300, and 400 °C, respectively, and the diffusion time was 2 h. The purpose of annealing on a hot plate inside the glovebox was to reduce the risks of Li oxidation, moisture absorption, and surface contamination during transfer and heating, thereby minimizing the influence of Li-layer instability under an open-air environment on the diffusion process. After diffusion, the sample was cooled to room temperature on the hot plate. The undiffused or residual Li layer on the surface was then removed by cleaning to reduce its interference with subsequent characterization, electrode deposition, and device testing. Specifically, the Li-diffused sample was handled under a dry inert atmosphere as much as possible. The residual Li layer was first gently removed from the surface using lint-free wipes, followed by rapid cleaning with anhydrous ethanol or anhydrous isopropanol. The sample was then dried with high-purity nitrogen and promptly transferred to the subsequent processing step. No water-based cleaning was used, and the exposure time of the Li-treated surface to ambient air was minimized to reduce the risk of Li oxidation, moisture adsorption, and surface contamination.

To improve the clarity and reproducibility of the contact-fabrication process, the main deposition and diffusion parameters used for Li and In contact formation are summarized in Table 1. The Li film was deposited at a low deposition rate to reduce excessive Li incorporation and near-surface instability. Because Li is chemically active, Li deposition and subsequent thermal diffusion were carried out under vacuum or inert-atmosphere protection as much as possible. In contrast, In diffusion required a higher thermal budget and was therefore performed in a three-zone tube furnace under an inert atmosphere.

#### 2.2.2. Preparation of In-Diffused Contacts

In diffusion was used to form a p^+^ surface diffusion contact region on the opposite side of the HPGe sample. Compared with Li, effective In diffusion in Ge requires higher thermal activation; therefore, solid-state thermal diffusion was carried out in a three-zone tube furnace. Specifically, after processing the Li-diffused side and removing the residual Li, a 200 nm In thin film was deposited by vacuum evaporation on the opposite side of the sample and used as the surface impurity source. The sample was then placed in the constant-temperature zone of a three-zone tube furnace and thermally diffused in an inert atmosphere for 8 h. The In diffusion temperatures were 600, 700, 800, and 900 °C, respectively. After diffusion, the sample was cooled to room temperature under a protective atmosphere, and the residual In layer on the surface was removed.

### 2.3. Electrode Preparation and Device Packaging

After Li and In diffusion treatments, Au electrodes with a thickness of 50 nm were deposited on the diffusion contact surfaces on both sides of the sample by vacuum evaporation. The Au electrodes were prepared using a high-vacuum resistance evaporation coating system. Solid Au pellets were used as the evaporation source. Approximately 1.0 g of Au pellets was loaded for each evaporation run. During deposition, the chamber pressure was maintained at approximately 2 × 10^−4^ Pa, and the substrate holder was rotated at 15 rpm to improve the uniformity of the electrode films. Solid Au pellets were used as the evaporation source. The evaporation current was approximately 96.6 A, and the deposition rate was maintained at approximately 0.3 Å s^−1^ until the Au thickness reached 50 nm. The Au electrodes were used to establish stable electrical connections between the diffusion contact layers and the external testing system, and served as the signal readout terminals for low-temperature I–V measurements and synchrotron X-ray I–T response tests. After electrode deposition, the sample was fixed in a testing fixture by wire connection, thereby obtaining a planar HPGe device with Li/In diffusion contacts. The effective device area was defined as the projected overlap region of the functional electrodes on the upper and lower sides and was used for subsequent comparison of dark current and X-ray response.

### 2.4. Structural, Chemical-State, and Optical Characterization

The crystal structure was characterized by X-ray diffraction (XRD, D/MAX-2200 V PC, Rigaku Corporation, Tokyo, Japan; Cu Kα radiation, λ = 0.15406 nm) to analyze the stability of the bulk Ge crystalline phase and any additional weak diffraction peaks that might appear after diffusion. Raman spectra were collected using a JY H800UV system (HORIBA Jobin Yvon S.A.S., Palaiseau, France) to characterize the position, full width at half maximum, and peak-shape evolution of the first-order optical phonon mode of Ge, thereby evaluating the near-surface lattice ordering, local strain, and structural disordering. The surface morphology was observed using field-emission scanning electron microscopy (FE-SEM, Sirion 200, FEI, Hillsboro, OR, USA) to analyze surface granulation, local agglomeration, and roughening behavior of HPGe before and after Li/In diffusion. Three-dimensional surface morphology and roughness were measured by atomic force microscopy (AFM, Dimension Icon, Bruker, Berlin, Germany), from which roughness parameters such as Rq and Ra were obtained. Surface chemical states were characterized by X-ray photoelectron spectroscopy (XPS, ESCALAB 250Xi, Thermo Scientific, Waltham, MA, USA), typically using a monochromatic Al Kα X-ray source. The depth distributions of Li and In were measured by secondary ion mass spectrometry (SIMS, Kore SurfaceSeer-I, Ely, UK) and compared with diffusion models. The in-plane elemental distribution and diffusion uniformity were characterized by micro-area X-ray fluorescence (μ-XRF) at the BL15U1 beamline of the Shanghai Synchrotron Radiation Facility. Low-temperature photoluminescence (PL) measurements at 77 K were performed using a fluorescence spectrometer system (FLS980, Edinburgh Instruments Ltd., Livingston, UK to analyze the influence of diffusion treatment on impurity–defect recombination behavior in the near-surface region of HPGe.

### 2.5. Low-Temperature Electrical Measurements and Synchrotron X-Ray Response Tests

Dark-current I–V measurements were performed at approximately 77 K to reduce the contribution of thermally generated intrinsic carriers in Ge to the dark current. During the measurements, a bias voltage was applied across the device, and the corresponding current response was recorded. Synchrotron X-ray current-response measurements were carried out at the BL15U1 beamline of the Shanghai Synchrotron Radiation Facility. BL15U1 is a hard X-ray microfocusing beamline that provides a high-flux monochromatic X-ray microbeam with tunable energy, making it suitable for micro-area X-ray fluorescence, absorption, diffraction, and detector-response measurements. X-ray I–T tests were performed by periodically switching the beam on and off and adjusting the incident photon flux to evaluate the response repeatability, net response current, and irradiated-to-dark current ratio of the device.

For data analysis, the structural, morphological, chemical, and electrical results were mainly used to compare the evolution trends among different diffusion temperatures. SEM, AFM, and μ-XRF measurements were checked at multiple surface regions, and representative images or maps are shown in the manuscript. The low-temperature I–V curves were obtained by repeated voltage sweeps under the same testing configuration, and the synchrotron X-ray response was evaluated using periodic beam on/off cycles to confirm the response repeatability. Therefore, the extracted values, including roughness, diffusion depth, and dark current, are used as comparative indicators for process-window evaluation.

### 2.6. Diffusion Model and Simulation Method

To evaluate the thermal diffusion depths of Li and In in HPGe, Sentaurus TCAD X-2025.09 (Synopsys, Inc., Sunnyvale, CA, USA) was used to calculate the impurity concentration profiles as a function of depth under different diffusion temperatures. The model used Ge as the matrix, with Li or In defined as the surface impurity source. Concentration–depth profiles were obtained according to the corresponding annealing temperature and duration. The simulation results were used to estimate the effective diffusion depths at different temperatures and were compared with the SIMS results. Because the actual diffusion profiles may deviate from ideal models due to the finite amount of thin-film source material, surface oxidation or residual layers, defect trapping, and crystal-defect-assisted diffusion, this work mainly compares the evolution trends of the diffusion front and the main diffusion region, while avoiding over-quantitative analysis of the deep low-concentration tail region.

## 3. Results and Discussion

### 3.1. Process Window for Li Diffusion

#### 3.1.1. Structural Stability and Near-Surface Morphological Evolution

**Figure 1:**
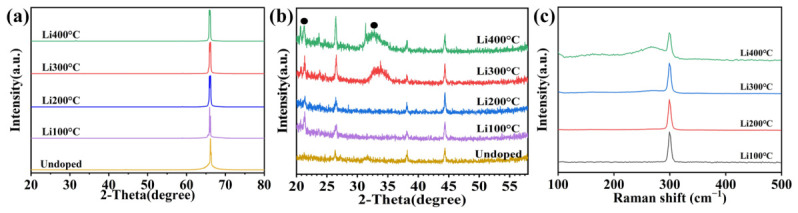
XRD and Raman spectra of HPGe samples after Li diffusion at different temperatures: (**a**) full-range XRD patterns from 20° to 80°; (**b**) enlarged XRD patterns from 20° to 59°; (**c**) Raman spectra.

Figure 1a shows the full-range XRD patterns of HPGe samples after Li diffusion at different temperatures. All samples exhibit a strong and sharp Ge(400) diffraction peak at around 2θ ≈ 66°, indicating that the HPGe matrix retains the diamond-cubic single-crystal Ge structure after Li diffusion. As the Li diffusion temperature increases from 100 °C to 400 °C, the Ge(400) main peak shows no obvious disappearance, severe broadening, or intensity attenuation, suggesting that Li diffusion within this temperature range does not cause overall damage to the bulk crystal structure. As shown in Figure 1b, weak peaks are already present in the undiffused sample at approximately 26.5°, 38.2°, and 44.4°. Considering that the sample was mounted in a hollowed aluminum holder and supported with plasticine during the measurement, the peaks at 38.2° and 44.4° are more likely to originate from the (111) and (200) diffraction background of the Al holder [21], whereas the peak near 26.5° may be associated with quartz/silicate fillers or carbonaceous components in the supporting material [22]. Since these peaks are already observed in the undiffused sample and do not show a systematic increase with Li diffusion temperature, they are not regarded here as evidence of Li-related new phases. In contrast, new weak peaks appear in the higher-temperature Li-diffused samples near 21.28° and 32.7–33.39°, which may be associated with surface reaction products such as Li_2_O, LiOH, Li_2_CO_3_, or Li–Ge–O compounds [23,24,25]. Figure 1c shows that the undiffused sample and the Li-diffused samples treated at 100–300 °C all exhibit a sharp first-order Ge optical phonon peak near 300 cm^−1^, indicating that low- to intermediate-temperature Li diffusion does not significantly disrupt the lattice ordering in the near-surface region of HPGe. This peak position is consistent with the characteristic first-order Raman mode of crystalline Ge reported in previous Raman studies [26]. In contrast, the 400 °C sample retains the Ge main peak but additionally shows a broadened vibrational band near 280 cm^−1^. Previous Raman studies of amorphous and disordered Ge have shown that broad Raman features in the 270–280 cm^−1^ region are commonly associated with amorphous-Ge-like Ge–Ge vibrational contributions, short-range structural disorder, and reduced crystalline ordering [27,28]. Raman studies of Ge nanostructures also indicate that phonon confinement and nanoscale structural disorder can lead to peak broadening and downshift of the Ge-related Raman mode [29,30]. Therefore, the broadened band observed in the 400 °C sample is interpreted as evidence of enhanced near-surface structural disorder, rather than as a definitive signature of a single Ge-related phase. Combined with the changes in the weak XRD peaks, these results indicate that Li diffusion at 400 °C exceeds the relatively mild contact-formation window and is likely accompanied by surface side reactions and local structural disordering.

**Figure 2:**
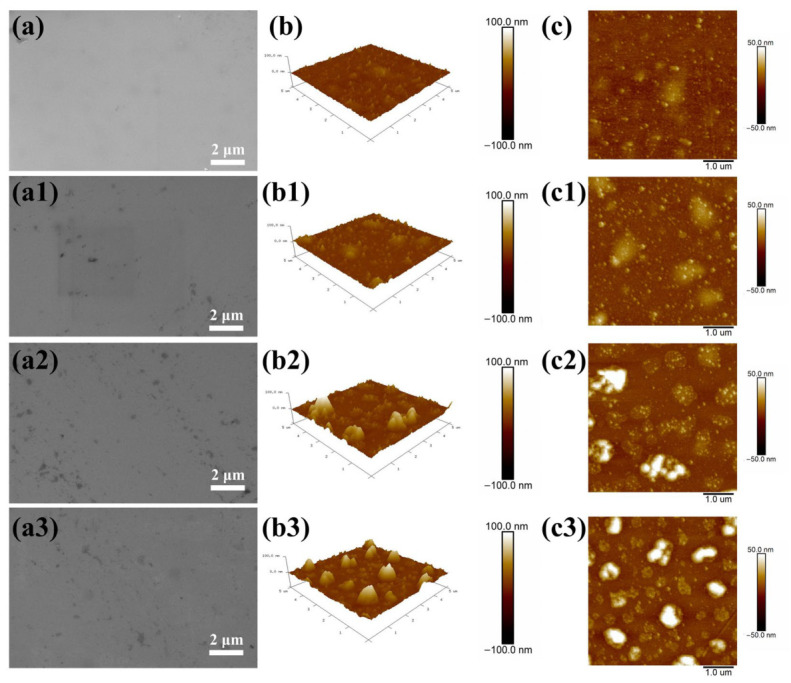
SEM and AFM surface morphology of HPGe samples after Li diffusion at different temperatures: (**a**–**a3**) SEM images; (**b**–**b3**) three-dimensional AFM morphology; (**c**–**c3**) two-dimensional AFM height maps. The four rows correspond to the undiffused sample and the samples diffused at 100 °C, 200 °C, and 300 °C, respectively.

Figure 2 further compares the surface morphology of the samples treated at different Li diffusion temperatures. The undiffused sample exhibits a smooth surface, with no obvious particle agglomeration or large-area corrosion features observed, and the Rq and Ra values are 3.98 nm and 2.77 nm, respectively. After diffusion at 100 °C, the surface remains continuous, although a small number of nanoscale protrusions appear, and the Rq and Ra values increase to 5.50 nm and 4.01 nm, respectively. For the 200 °C sample, the number of dark particles and locally nonuniform regions increases, and the Rq and Ra values rise to 11.10 nm and 6.73 nm, respectively. In the 300 °C sample, the granular features are further enhanced, with more pronounced island-like protrusions and local height fluctuations, and the Rq and Ra values reach 12.50 nm and 8.18 nm, respectively.

These results indicate that increasing the Li diffusion temperature continuously enhances the surface roughening of HPGe. This morphology evolution may be related to thermally activated Li migration on the Ge surface, the formation of local Li-containing reaction products, and the aggregation of residual Li-related particles. Surface diffusion of Li adatoms on Ge(100) and Ge(111) surfaces has been experimentally reported, and Li diffusion in Ge during heat treatment has also been used for n^+^ contact formation in Ge-based detectors [31,32,33]. For diffusion contacts, moderate diffusion is beneficial for the formation of an n^+^ contact layer, whereas excessive diffusion may increase the surface-state density and local electric-field distortion, thereby raising the risk of non-ideal leakage current. Therefore, the Li diffusion temperature must be balanced between contact-layer formation and surface morphological stability.

#### 3.1.2. Surface Chemical States and In-Plane Uniformity

**Figure 3:**
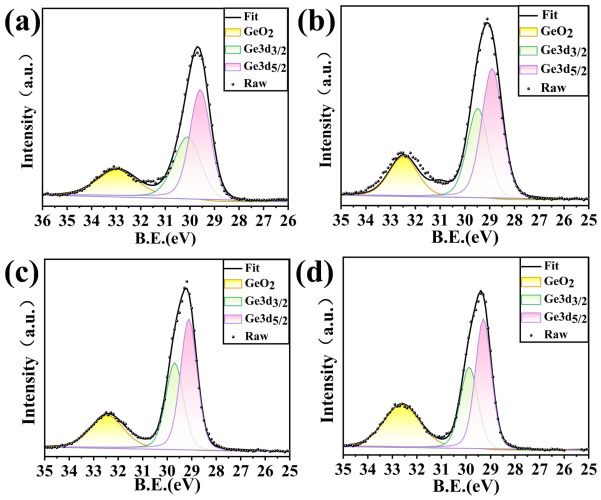
High-resolution Ge 3d XPS spectra of the undiffused HPGe sample and the samples after Li diffusion at different temperatures: (**a**) undiffused sample; (**b**) 100 °C; (**c**) 200 °C; (**d**) 300 °C.

Figure 3 shows the high-resolution Ge 3d XPS spectra of the undiffused HPGe sample and the samples after Li diffusion at different temperatures. The Ge 3d spectra of all samples can be deconvoluted into the 3d_5/2_ and Ge 3d_3/2_ doublet of metallic/crystalline Ge^0^, together with GeO_2_-related components located on the high-binding-energy side. The Ge^0^ doublet mainly originates from Ge–Ge bonding in the near-surface region of the HPGe matrix, whereas the high-binding-energy peak near 32–33 eV corresponds to the Ge^4+^ oxidation state, indicating that native oxide or diffusion-induced oxide products exist on the sample surface. GeO_2_/Ge interface studies have shown that oxide quality, defect passivation, and chemical treatment strongly affect interfacial states and electrical stability [34,35,36,37,38]. For the undiffused sample, the Ge^0^ doublet is dominant, while the GeO_2_ peak is relatively weak, suggesting that the surface-treated HPGe sample is mainly characterized by the crystalline Ge matrix signal, with only a thin native oxide layer at the outermost surface. As shown in Figure 3b, after Li diffusion at 100 °C, the Ge^0^ main peak remains clear, but the GeO_2_ component on the high-binding-energy side becomes more pronounced. This result indicates that even at relatively low temperature, the deposited Li layer and the subsequent thermal diffusion process can modify the HPGe surface chemical environment. The peak position of the Ge^0^ 3d_5/2_ component also changes after Li diffusion. For the undiffused, 100 °C, 200 °C, and 300 °C samples, the fitted Ge^0^ 3d_5/2_ peak positions are approximately 29.5, 28.9, 29.1, and 29.3 eV, respectively. The shift to lower binding energy after Li diffusion may be associated with Li-induced modification of the near-surface electronic environment, such as changes in local surface potential or band bending. With increasing diffusion temperature, the Ge^0^ 3d_5/2_ peak gradually shifts back toward higher binding energy, accompanied by a more pronounced high-binding-energy GeO_2_-related component. Previous high-resolution XPS studies of oxidized Ge surfaces have shown that Ge–O bonding and different Ge oxidation states can induce positive chemical shifts in Ge 3d spectra, while Ge/oxide interface states can also lead to band bending and binding-energy shifts [39,40,41]. Therefore, the observed Ge 3d peak shift is interpreted as the combined result of Li-induced near-surface electronic modification, surface oxidation, and interfacial chemical-state changes, rather than as evidence of a single chemical process. Because Li is highly chemically active, residual Li or Li-related surface layers may readily react with trace O_2_/H_2_O during diffusion, cooling, and transfer, thereby promoting surface oxidation or the formation of Li–Ge–O-related transition layers. Ozone and thermal oxidation studies of Ge surfaces further support the sensitivity of Ge oxidation kinetics to processing conditions [42]. When the diffusion temperature increases to 200 °C and 300 °C, the Ge^0^ doublet maintains a well-defined peak shape, indicating that the near-surface region of HPGe after Li diffusion retains crystalline Ge–Ge bonding without obvious overall chemical decomposition, as shown in Figure 3c,d. However, the GeO_2_ component remains present, consistent with the surface granulation and increased roughness observed by SEM/AFM. This suggests that higher-temperature Li diffusion enhances surface reactions and local oxidation. To further verify the presence of Li-related components near the surface after Li diffusion, high-resolution Li 1s XPS measurements were performed on the samples diffused at different temperatures, as shown in Figure S1. Broad Li 1s-related peaks can be observed in the range of approximately 54–55 eV for the diffused samples, indicating that Li has been introduced into the near-surface region of HPGe. Because the Li 1s signal is weak and the binding energies of different Li chemical states are close to each other, no specific phase assignment is made in this work; instead, the Li 1s signal is used as auxiliary evidence for the presence of Li-related surface species.

**Figure 4:**
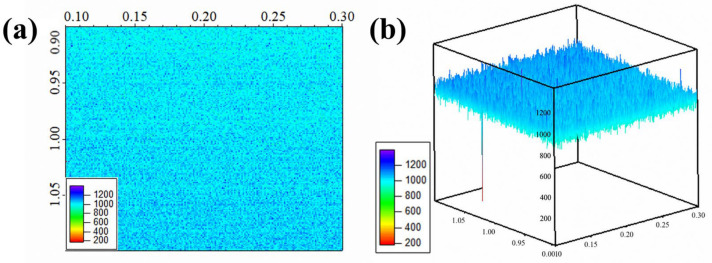
Synchrotron micro-area X-ray fluorescence maps of the HPGe sample after Li diffusion at 200 °C: (**a**) two-dimensional fluorescence intensity map; (**b**) three-dimensional fluorescence intensity distribution.

Figure 4 shows the two-dimensional micro-area X-ray fluorescence map of the HPGe sample after Li diffusion at 200 °C, obtained at the BL15U1 beamline of the Shanghai Synchrotron Radiation Facility. The 200 °C sample was selected as a representative Li-diffused sample to evaluate the in-plane uniformity under a moderate diffusion condition. As shown in Figure 4a, the fluorescence intensity is uniform overall across the sample surface, with no obvious large-area enrichment, depletion, or stripe-like nonuniform distribution. The three-dimensional intensity distribution in Figure 4b further shows that the fluorescence signal fluctuates only slightly around the average level, with only a few local spikes or low-intensity points. These features may be related to small surface particles, local roughness variations, statistical noise, or micro-area absorption differences. Li is a low-atomic-number light element with extremely low characteristic X-ray energy; therefore, conventional hard X-ray μ-XRF measurements cannot directly detect Li. The result is therefore interpreted as evidence for the overall surface-response uniformity of the selected diffusion condition rather than as a direct Li map.

**Figure 5:**
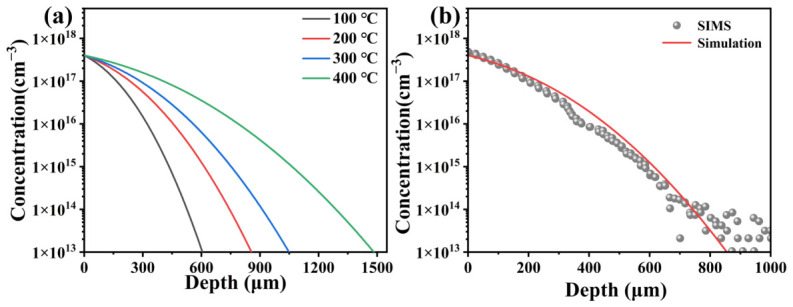
Diffusion simulation and SIMS depth profiles of Li in HPGe: (**a**) simulated Li concentration–depth profiles at different temperatures under infinite-source diffusion conditions; (**b**) SIMS concentration–depth profiles of the Li-diffused samples and comparison with the diffusion model.

Figure 5a shows the simulated Li concentration–depth profiles in Ge at different temperatures under infinite-source diffusion conditions. As the diffusion temperature increases from 100 °C to 400 °C, the Li concentration decay curves gradually shift toward greater depths, and the diffusion front becomes significantly deeper. When the Li concentration decreases to approximately 10^13^ cm^−3^, this value is used as the criterion for the effective diffusion depth. Accordingly, the diffusion depths at 100 °C, 200 °C, 300 °C, and 400 °C are approximately 606.7, 856.6, 1049.9, and 1483.5 μm, respectively. These results indicate that temperature is a key factor controlling Li diffusion depth. Increasing the diffusion temperature can significantly increase the diffusion distance of Li in Ge and form a thicker n-type diffusion layer. Figure 5b shows the SIMS concentration–depth profiles of the Li-diffused samples and their comparison with the diffusion model. The measured results show that the Li concentration reaches its maximum at the outermost surface and then decreases rapidly in the shallow region, indicating pronounced Li enrichment near the surface. At greater depths, the concentration decreases more gradually and exhibits a long low-concentration diffusion tail, demonstrating that Li is not confined to the surface but effectively diffuses into the Ge bulk. Previous studies on dopant diffusion, process simulation, and carrier profiling in Ge have shown that measured dopant profiles can deviate from ideal diffusion models because of defect coupling, dopant deactivation, non-equilibrium boundary conditions, and measurement sensitivity limits [13,14,17,18,43]. The simulated curves agree reasonably well with the SIMS results in the high-concentration surface region and the main diffusion region, indicating that the diffusion model used in this work can describe the primary diffusion behavior of Li in HPGe. The main differences appear in the deep low-concentration tail region, where the measured profiles show a more gradual decay and a longer tail than the ideal model. This may be related to a non-constant Li source during diffusion, Li trapping by near-surface defects or oxide layers, crystal-defect-assisted diffusion, and signal fluctuations as the SIMS measurement approaches its detection limit. Therefore, the SIMS results not only verify Li diffusion-layer formation, but also indicate that actual Li diffusion is more complex than the ideal model, especially in the low-concentration tail region.

### 3.2. Process Window for in Diffusion

#### Structural Stability and Surface Roughening Behavior

**Figure 6:**
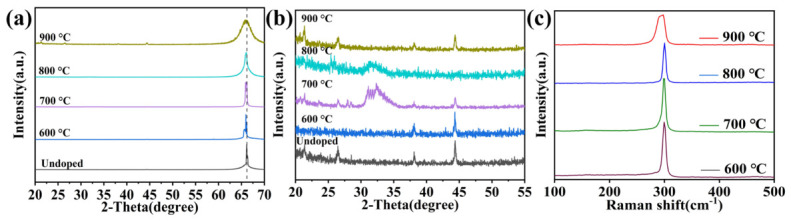
Structural characterization of HPGe samples after In diffusion at different temperatures: (**a**) full-range XRD patterns from 20° to 70°; (**b**) enlarged XRD patterns from 20° to 55°; (**c**) Raman spectra.

Figure 6a,b shows the XRD patterns of the undiffused HPGe sample and the samples after In diffusion at different temperatures. All samples exhibit a strong diffraction peak at around 2θ ≈ 66°, corresponding to the (400) plane of diamond-cubic Ge, indicating that the bulk crystal structure of HPGe is retained after In diffusion at 600–900 °C, without obvious bulk decomposition or formation of a new dominant phase. To more clearly show the weak diffraction features in the low-angle region, the enlarged XRD patterns from 20° to 55° are provided separately in Figure 6b. In the enlarged patterns, weak diffraction features can be observed near 21.4°, 26.5°, 38.1–38.2°, and 44.3–44.4° in the undiffused and In-diffused samples. Similar to the discussion for the Li-diffused samples, the peaks near 38.1–38.2° and 44.3–44.4° are close to the diffraction background of the Al holder, while the weak peak near 26.5° may be related to quartz/silicate-containing supporting materials or other background contributions [44]. Since these peaks are already present in the undiffused sample or do not show a monotonic increase with In diffusion temperature, they are not regarded as direct evidence of In-related new phases. It is also noted that the samples diffused at 700 and 800 °C show relatively more pronounced weak features around 32–33° compared with the undiffused and 600 °C samples. This angular region is close to the main diffraction feature of tetragonal metallic In reported for thermally grown In films [45]. Therefore, these weak features may be associated with residual In-related species. In addition, In-O or Ge-In/O-related near-surface reaction products cannot be excluded, because indium oxide and indium germanate phases can form under thermal treatment and oxygen-involved conditions [46,47]. However, because these peaks are weak, partially overlapped with background signals, and do not show a simple temperature-dependent evolution, they are used only as auxiliary evidence for possible near-surface reactions rather than as definitive phase identification. In addition, the Ge(400) peak of the 900 °C sample is noticeably broadened, suggesting that excessive diffusion temperature may induce near-surface structural disorder, thermal-stress accumulation, or the formation of a surface reaction layer.

Figure 6c shows the Raman spectra of the samples after In diffusion at different temperatures. The samples diffused at 600–800 °C all exhibit a sharp first-order Ge optical phonon peak near 300 cm^−1^, indicating that the Ge–Ge lattice vibration characteristics are preserved within this temperature range and that near-surface lattice ordering is not significantly disrupted. In contrast, the Raman peak of the 900 °C sample shifts to approximately 294.8 cm^−1^ and is accompanied by noticeable peak broadening. Stress-induced shifts in first-order Raman modes in Ge and other diamond-type semiconductors have been reported, indicating that Raman peak positions are sensitive to local strain and residual stress [48]. In polycrystalline Ge films, strain variation and structural disorder can also lead to Raman peak shift, broadening, and intensity variation [49]. In addition, the phonon confinement model shows that reduced coherent scattering length or microstructural disorder can broaden and shift the one-phonon Raman spectra of crystalline semiconductors [50]. Similar strain-related optical responses have also been discussed for amorphous Ge thin films [51]. Therefore, the red shift and broadening observed for the 900 °C sample may be related to thermal-stress relaxation, residual strain, In-diffusion-induced lattice perturbation, and increased near-surface structural disorder. Therefore, although 900 °C is beneficial for increasing In diffusion depth, it significantly increases structural and interfacial instability.

**Figure 7:**
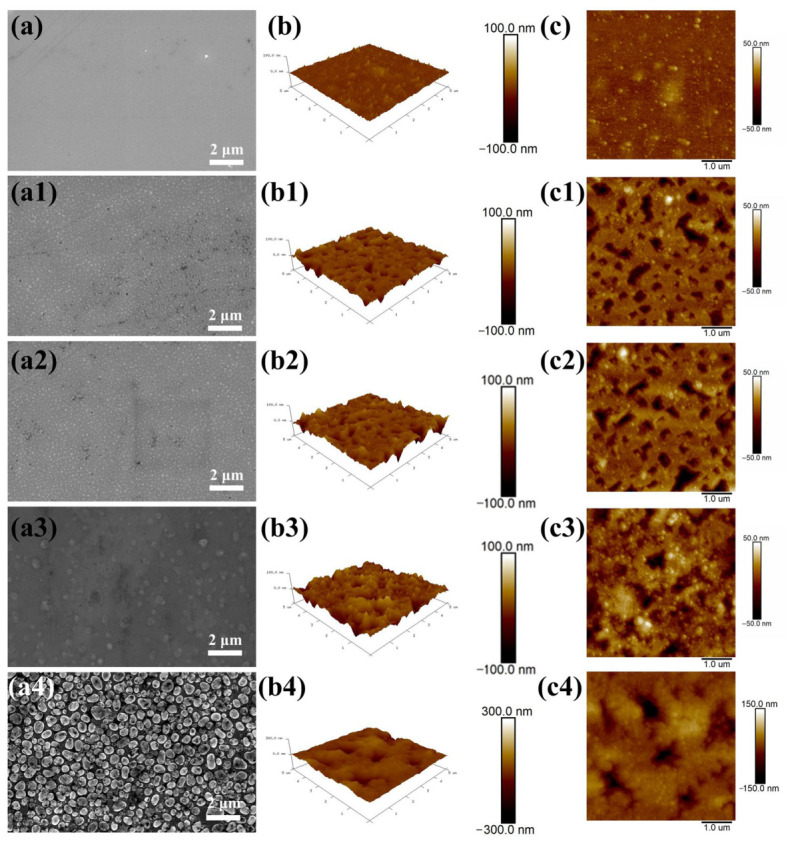
SEM and AFM surface morphology of HPGe samples after In diffusion at different temperatures: (**a**–**c**) undiffused sample; (**a1**–**c1**) 600 °C; (**a2**–**c2**) 700 °C; (**a3**–**c3**) 800 °C; (**a4**–**c4**) 900 °C.

Figure 7 presents the surface morphology of the undiffused HPGe sample and the samples after In diffusion at different temperatures. The left column shows the SEM images, while the right panels show the three-dimensional AFM morphology and two-dimensional height maps. The undiffused sample exhibits a relatively flat surface, with only a small number of fine particles and local height fluctuations. The Rq and Ra values are 3.98 nm and 2.77 nm, respectively, indicating that the initial HPGe surface has good flatness. After In diffusion at 600 °C, uniformly distributed fine particles and local protrusion/depression structures appear on the sample surface. The Rq and Ra values increase to 10.8 nm and 7.67 nm, respectively, indicating that In diffusion has already induced noticeable near-surface morphological perturbation. As the diffusion temperature increases to 700 °C and 800 °C, surface granulation and local height fluctuations are further enhanced. However, the increase in roughness remains relatively limited, with Rq/Ra values of 12.0/8.37 nm and 12.1/9.24 nm, respectively. This suggests that In diffusion in the range of 600–800 °C causes HPGe surface roughening, but the overall morphological degradation remains relatively controllable. In contrast, the sample diffused at 900 °C exhibits significant particle agglomeration and island-like structure formation. A large number of micrometer-scale particles cover the surface in the SEM image, and the AFM height fluctuations increase markedly. The Rq and Ra values sharply rise to 30.1 nm and 19.4 nm, respectively. Such morphology evolution is consistent with reports showing that Ge surface topography can be strongly modified by processing-induced roughening, oxidation, and energetic or thermal surface restructuring [42,52]. Combined with the XRD and Raman results, these observations indicate that In diffusion at 600–800 °C can introduce surface diffusion modification while maintaining relative bulk structural stability. In contrast, high-temperature diffusion at 900 °C causes pronounced surface roughening and near-surface structural perturbation. Therefore, the In diffusion temperature should not be excessively high, and 700–800 °C is more suitable as the process window for subsequent p^+^ diffusion contact preparation.

Figure S2 shows the high-resolution Ge 3d XPS spectra of the samples after In diffusion at 600–900 °C. All spectra can be deconvoluted into Ge^0^ 3d_5/2_ and Ge^0^ 3d_3/2_ components, together with GeO_2_-related components on the high-binding-energy side. The Ge^0^ doublet originates from Ge–Ge bonding in the near-surface region of the HPGe matrix, whereas the GeO_2_ component indicates the presence of a native oxide layer or oxide products formed during diffusion. As the In diffusion temperature changes, the GeO_2_ component remains relatively pronounced, indicating that high-temperature In diffusion and subsequent air exposure readily lead to Ge surface oxidation. This interpretation is consistent with studies showing that GeO_2_/Ge interface quality, oxide removal, and passivation chemistry can strongly affect interfacial defect density and surface stability [34,35,36,37,38]. It should be noted that the probing depth of XPS is shallow; therefore, the enhanced GeO_2_ signal mainly reflects the oxidation state of the outermost surface and does not imply oxidative decomposition of the HPGe bulk structure. Figure S3 shows the high-resolution In 3d XPS spectra of the samples after In diffusion at 600–900 °C. Clear In 3d_5/2_ and In 3d_3/2_ spin–orbit doublets are observed for samples treated at different diffusion temperatures, indicating that In-related components remain in the near-surface region of HPGe after vacuum evaporation and thermal diffusion. The intensity and peak shape of the In 3d doublet vary with diffusion temperature, which may reflect the combined effects of In retention, inward diffusion, surface segregation, and temperature-dependent surface reaction states. Previous XPS studies have shown that In 3d core-level spectra of metallic In and In-containing compounds can exhibit close binding energies and subtle peak-shape differences, making unambiguous chemical-state assignment based only on the In 3d region difficult [53]. In addition, the surface speciation of In and indium oxides can be modified by exposure to atmospheric oxidants such as O_2_ and H_2_O, leading to changes in oxide- and hydroxide-related surface species [54]. Therefore, the temperature-dependent changes in the In 3d spectral intensity and peak shape are interpreted here as evidence of changes in near-surface In retention and reaction state, rather than as definitive identification of a single In-containing phase. 

Figure S4 shows the two-dimensional fluorescence maps and three-dimensional intensity distributions of the HPGe sample after In diffusion at 600 °C. The 600 °C sample was selected as a representative low-temperature In-diffused sample to evaluate the in-plane uniformity of the detectable In-related fluorescence signal under a relatively mild diffusion condition. In Figure S4a,b, the high-intensity fluorescence signal is relatively uniform overall, with no obvious large-area enrichment or depletion regions, indicating good in-plane consistency of the detectable elemental signal on the sample surface. In Figure S4c,d, the low-intensity fluorescence signal mainly appears as randomly distributed weak points and local spikes. This behavior may be related to the low intensity of the detected In-related signal, statistical fluctuations, surface roughness variations, or isolated microparticles. In X-ray spectral imaging, low photon counts can introduce pronounced Poisson noise and baseline fluctuations, which may affect the reliability of weak-intensity features [55]. In addition, micro-XRF maps can be influenced by fluorescence noise, spatial-resolution limitations, point-spread-function effects, and positioning or focusing uncertainties [56]. Therefore, the randomly distributed weak points in the low-intensity fluorescence map are not regarded as evidence of large-scale elemental segregation. Overall, no obvious large-scale elemental segregation or severe local enrichment is observed in the sample diffused at 600 °C, indicating that the surface composition distribution remains relatively uniform under low-temperature In diffusion conditions.

**Figure 8:**
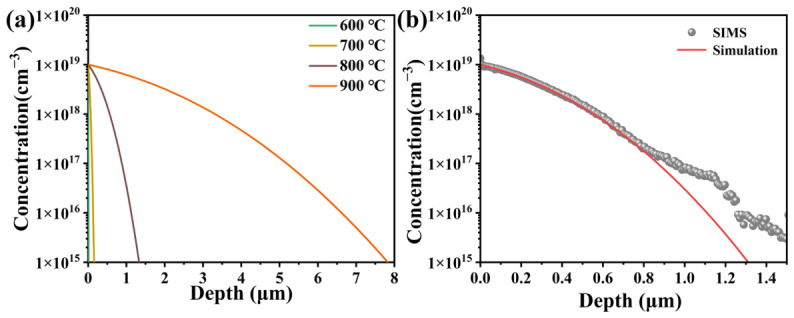
Diffusion simulation and SIMS depth profiles of In in HPGe: (**a**) simulated In concentration–depth profiles at different temperatures under infinite-source diffusion conditions; (**b**) SIMS concentration–depth profiles of the In-diffused samples and comparison with the diffusion model.

Figure 8a shows the simulated In concentration–depth profiles in HPGe at different temperatures under infinite-source diffusion conditions. The results indicate that In diffusion depth is extremely sensitive to temperature. As the diffusion temperature increases from 600 °C to 900 °C, the In concentration decay curves shift markedly toward greater depths. When the In concentration decreases to approximately 10^15^ cm^−3^, this value is used as the criterion for the effective diffusion depth. Accordingly, the diffusion depths at 600 °C, 700 °C, 800 °C, and 900 °C are approximately 0.01, 0.15, 1.33, and 7.83 μm, respectively. These results indicate that below 700 °C, In is mainly confined to an extremely shallow surface region, whereas above 800 °C, the In diffusion depth increases significantly. This demonstrates that high-temperature thermal activation is a key factor for forming an effective p^+^ diffusion layer. Figure 8b shows the SIMS concentration–depth profiles of the In-diffused samples and their comparison with the diffusion model. The measured results show that the In concentration is relatively high in the near-surface region, on the order of 10^19^ cm^−3^, and then gradually decreases with increasing depth, indicating that In has effectively diffused from the surface into the near-surface region of HPGe. Studies of dopant diffusion and activation in Ge, including In diffusion modeling, show that dopant profiles can be governed by defect-mediated diffusion, activation/deactivation behavior, and carrier concentration effects [14,15,16,43,57,58]. The simulated curves agree reasonably well with the SIMS results in the shallow surface region and the main diffusion region, suggesting that the infinite-source diffusion model can describe the primary diffusion behavior of In in Ge. The main differences appear in the deeper region, where the SIMS profiles show slower decay and a longer low-concentration tail than the ideal model. This may be related to a non-constant surface In source, In trapping by near-surface defects or oxide layers, crystal-defect-assisted diffusion, and quantitative uncertainty in the low-concentration region of SIMS measurements. Overall, In diffusion depth increases significantly with temperature, but excessive temperature also causes surface roughening and near-surface structural perturbation. Therefore, In diffusion temperature must be balanced between obtaining sufficient p^+^ diffusion depth and maintaining surface/interfacial stability.

### 3.3. Low-Temperature Dark Current and X-Ray Response

**Figure 9:**
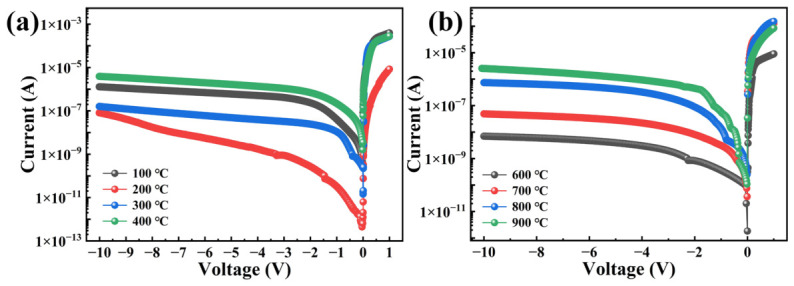
Dark-current I–V characteristics of HPGe devices prepared at different diffusion temperatures: (**a**) Li-diffused samples; (**b**) In-diffused samples.

Figure 9 shows the dark-current I–V characteristics of the Li-diffused and In-diffused HPGe devices prepared at different diffusion temperatures. As shown in Figure 9a, the Li diffusion temperature has a pronounced influence on device dark current. Under a reverse bias of −10 V, the dark currents of the samples diffused at 100 °C, 200 °C, 300 °C, and 400 °C are 1.28 × 10^−6^, 8.07 × 10^−8^, 1.60 × 10^−7^, and 3.88 × 10^−6^ A, respectively. The sample diffused at 200 °C exhibits the lowest dark current, indicating that moderate Li diffusion is beneficial for forming a stable n^+^ contact layer and suppressing leakage current. The relatively high dark current of the 100 °C sample may be related to insufficient Li diffusion and incomplete contact-layer formation. In contrast, the significant increase in dark current for the 400 °C sample is consistent with the surface reactions, roughening, and near-surface disordering observed in the XRD, Raman, and AFM results, suggesting that excessively high Li diffusion temperature introduces more surface states, local defects, and non-ideal leakage paths. Therefore, Li diffusion is not simply improved by increasing temperature; instead, an optimized process window exists. Figure 9b shows the dark-current I–V curves of the samples prepared at different In diffusion temperatures. At −10 V, the dark currents of the samples diffused at 600 °C, 700 °C, 800 °C, and 900 °C are 6.96 × 10^−9^, 4.87 × 10^−8^, 7.41 × 10^−7^, and 2.54 × 10^−6^ A, respectively, showing an overall increasing trend with increasing diffusion temperature. The 600 °C sample exhibits the lowest dark current, indicating that lower In diffusion temperature leads to fewer surface-damage-related and thermally induced leakage paths. As the temperature increases, especially in the range of 800–900 °C, the dark current increases significantly, indicating that high-temperature In diffusion aggravates surface roughening, oxidation, and near-surface structural perturbation, thereby enhancing surface leakage and local injection current. These trends are consistent with studies showing that Ge detector leakage current and low-temperature contact behavior are strongly affected by contact barriers, amorphous interlayers, and contact-induced surface states [59,60]. Combined with the significant granulation and sharp increase in roughness observed for the 900 °C sample in Figure 7, these results indicate that excessively high In diffusion temperature is unfavorable for fabricating low-leakage devices. Overall, the Li-diffused sample exhibits a relatively low dark current near 200 °C, whereas the dark current of the In-diffused samples gradually increases with increasing diffusion temperature. These results demonstrate that the diffusion-contact process requires a balance between diffusion-layer formation and surface/interfacial stability: insufficient diffusion results in incomplete contact-layer formation, whereas excessive thermal budget causes surface reactions, morphological degradation, and increased defect-assisted leakage.

To provide a clearer comparison of the temperature-dependent trends, the key roughness, simulated diffusion depth, dark current, and process indication values are summarized in Table 2. Table 2 further highlights the competing effects of diffusion temperature. Moderate diffusion improves contact formation and suppresses dark current, whereas excessive thermal budget increases surface roughening, near-surface disordering, and leakage-current paths. Therefore, the optimized diffusion process should be determined by balancing diffusion depth with surface and interfacial stability.

**Figure 10:**
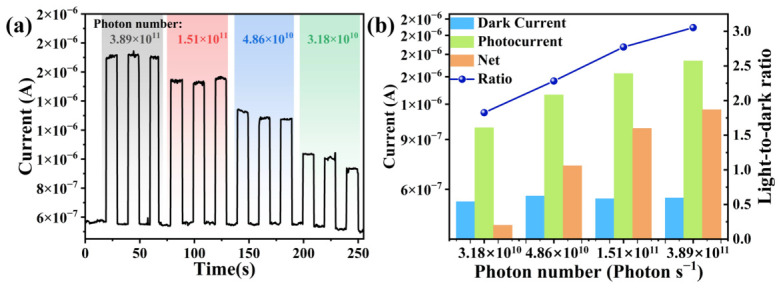
X-ray current response of the HPGe device with Li/In diffusion contacts under different incident photon fluxes. (**a**) I–T response curves under periodic X-ray on/off switching; (**b**) dark current, irradiated current, net response current, and photo-to-dark current ratio.

Figure 10a shows the X-ray current response curves of the HPGe device with Li/In diffusion contacts under different incident photon fluxes. During periodic switching of the X-ray beam on and off, the device current rapidly and reproducibly switches between the dark-current level and the irradiated-current level, demonstrating a stable current response to synchrotron X-rays. Under different photon fluxes, the dark current remains essentially within the range of approximately 5.4 × 10^−7^–5.7 × 10^−7^ A, indicating that the device baseline is relatively stable during measurement. Therefore, the variation in response current mainly originates from changes in incident X-ray photon flux rather than dark-current drift. The extracted results in Figure 10b show that, as the incident photon flux increases from 3.18 × 10^10^ photon s^−1^ to 3.89 × 10^11^ photon s^−1^, the average irradiated current increases from 9.90 × 10^−7^ A to 1.71 × 10^−6^ A, the average net response current increases from 4.48 × 10^−7^ A to 1.15 × 10^−6^ A, and the photo-to-dark current ratio increases from 1.825 to 3.053. This result indicates that increasing incident photon flux generates more electron–hole pairs, thereby enhancing photogenerated current and net response signal. It should be noted that the net response current increases with photon flux but does not show strict linear growth. This behavior may be related to carrier recombination, trap capture, space-charge effects, or changes in charge-collection efficiency under high photon flux. Recent Ge detector studies have emphasized the importance of charge-collection behavior, event selection, and X-ray spectrometric response when interpreting detector signals under low-energy or high-rate conditions [61,62,63]. Overall, Figure 10 confirms a clear and repeatable X-ray photoconductive response of the HPGe device with Li/In diffusion contacts under low bias. The response intensity increases as the incident photon flux increases.

### 3.4. Low-Temperature PL and Diffusion-Induced Recombination Mechanism

**Figure 11:**
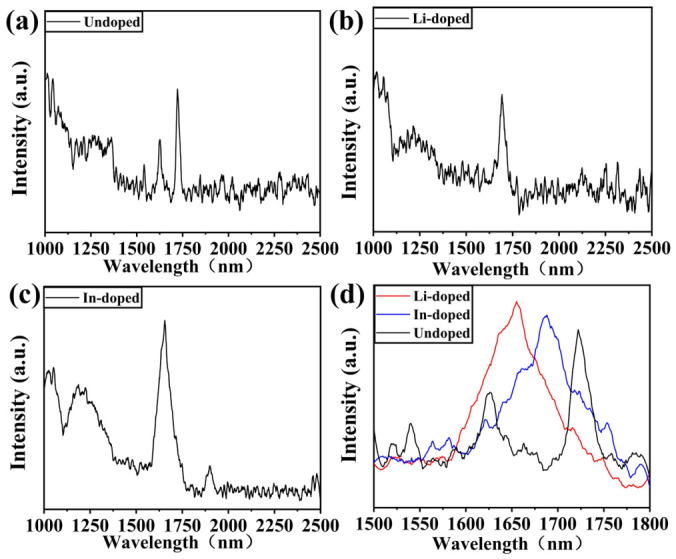
Low-temperature PL spectra of the undiffused and Li/In-diffused HPGe samples at 77 K. (**a**) Undiffused sample; (**b**) Li-diffused sample; (**c**) In-diffused sample; (**d**) comparison of normalized PL spectra.

Figure 11 shows the low-temperature PL spectra of the undiffused, Li-diffused, and In-diffused HPGe samples measured at 77 K. The undiffused sample exhibits a distinct narrow emission peak in the near-infrared region, with the main peak located at approximately 1720 nm, together with a weak emission feature on the shorter-wavelength side. This indicates that the pristine HPGe sample is dominated mainly by near-band-edge recombination of Ge and a small amount of impurity-/defect-related recombination at low temperature. The relatively narrow peak shape also suggests that the near-surface lattice integrity of the undiffused sample is relatively good, with fewer nonradiative recombination centers. After Li diffusion, the PL spectrum changes from a narrow peak to a broader emission band. After normalization, the main emission center is located at approximately 1650 nm, showing an obvious blue shift and peak broadening compared with the undiffused sample. This indicates that Li diffusion not only introduces donor-type impurities and changes the near-surface carrier concentration and compensation state, but also induces variations in local strain, impurity–defect recombination centers, and band-tail states. Studies of Ge optical behavior and surface-oxide effects indicate that band-edge emission and defect-related luminescence can be strongly affected by surface condition, oxide formation, and passivation [64,65,66]. After Li enters p-type Ge as a shallow donor, it can form an n^+^ diffusion layer and modify near-surface band bending. As a result, the low-temperature radiative recombination process changes from the original near-band-edge recombination to recombination involving Li-related shallow donors, defect states, or band-tail states, leading to a broadened emission band. The In-diffused sample also exhibits pronounced emission broadening, with the main peak located at approximately 1680–1690 nm, between those of the Li-diffused and undiffused samples. As an acceptor impurity, In forms a p^+^ diffusion region in the near-surface region of Ge, introducing acceptor-related energy levels and local lattice perturbation. Meanwhile, surface roughening, oxide layers, and local defects generated during high-temperature In diffusion may also increase near-surface recombination channels, resulting in PL peak broadening and peak-position variation. Compared with the Li-diffused sample, the emission peak of the In-diffused sample is closer to the longer-wavelength region, indicating that the two diffusion impurities regulate the near-surface band structure, compensation state, and defect-related recombination pathways in different ways. Therefore, the shifts in PL peak position and the broadening of the PL spectra after Li/In diffusion indicate that diffusion treatment significantly modifies the impurity–defect recombination environment in the near-surface region of HPGe. This result is consistent with the Raman, XPS, and AFM results, suggesting that the formation of diffusion contact layers is not a simple impurity-injection process, but is accompanied by coupled reconstruction of near-surface structure, chemical state, and defect-related recombination pathways.

## 4. Conclusions

In this work, Li/In double-sided diffusion contacts were prepared on p-type HPGe single crystals by vacuum evaporation of thin-film sources followed by solid-state thermal diffusion, and the effects of diffusion temperature on near-surface structure, morphology, diffusion behavior, and device response were investigated. The results show that moderate Li diffusion can form an effective n^+^ contact while maintaining the stability of the bulk Ge structure. The sample diffused at 200 °C exhibits the lowest dark current, 8.07 × 10^−8^ A at −10 V, whereas excessively high Li diffusion temperature induces surface reactions, roughening, and near-surface disordering. For In diffusion, the surface morphology and structure remain relatively controllable in the range of 600–800 °C, whereas diffusion at 900 °C leads to pronounced particle agglomeration, Raman peak red shift, and increased dark current, indicating that an excessive thermal budget is unfavorable for forming low-leakage contacts. SIMS measurements and TCAD simulations confirm that both Li and In can effectively diffuse into HPGe, while the measured profiles exhibit low-concentration tailing, reflecting the influence of defect trapping and defect-assisted diffusion. The final device with Li/In diffusion contacts shows a stable photoconductive response under synchrotron X-ray irradiation. As the photon flux increases, the net response current increases from 4.48 × 10^−7^ A to 1.15 × 10^−6^ A, and the photo-to-dark current ratio increases from 1.825 to 3.053. This work demonstrates that optimizing Li/In double-sided diffusion contacts in HPGe requires balancing diffusion-layer formation with near-surface and interfacial stability, rather than simply increasing diffusion temperature. Compared with studies focusing on a single contact type or a single performance parameter, this study establishes a coupled process–structure–diffusion–device relationship for evaporated thin-film-source Li/In contacts and identifies the main failure boundary caused by excessive thermal budget.

## Figures and Tables

**Table 1:** Deposition and diffusion parameters for Li and In contact formation.

Parameter	Li-Diffused Contact	In-Diffused Contact
Contact type	n^+^ contact layer	p^+^ surface contact region
Evaporation source	Solid Li foil	Solid In pellets
Loaded source mass	At least approximately 0.25 g	At least approximately 1.0 g
Deposition method	Vacuum thermal evaporation	Vacuum thermal evaporation
Target film thickness	100 nm	200 nm
Deposition rate	0.5 Å s^−1^	Approximately 0.3 Å s^−1^
Chamber pressure during deposition	Approximately 2 × 10^−4^ Pa	Approximately 0.1 Pa
Diffusion equipment	Hot plate in an inert-atmosphere glovebox	Three-zone tube furnace
Diffusion atmosphere	Argon atmosphere	Argon atmosphere
Diffusion temperature	100, 200, 300, and 400 °C	600, 700, 800, and 900 °C
Diffusion time	2 h	8 h

**Table 2:** Summary of key process-dependent parameters for Li- and In-diffused HPGe samples.

Diffusion Type	Temperature	Surface Roughness Rq/Ra	Simulated Diffusion Depth	Dark Current at −10 V
Li diffusion	100 °C	5.50/4.01 nm	606.7 μm	1.28 × 10^−6^ A
Li diffusion	200 °C	11.10/6.73 nm	856.6 μm	8.07 × 10^−8^ A
Li diffusion	300 °C	12.50/8.18 nm	1049.9 μm	1.60 × 10^−7^ A
Li diffusion	400 °C	Not measured by AFM	1483.5 μm	3.88 × 10^−6^ A
In diffusion	600 °C	10.8/7.67 nm	0.01 μm	6.96 × 10^−9^ A
In diffusion	700 °C	12.0/8.37 nm	0.15 μm	4.87 × 10^−8^ A
In diffusion	800 °C	12.1/9.24 nm	1.33 μm	7.41 × 10^−7^ A
In diffusion	900 °C	30.1/19.4 nm	7.83 μm	2.54 × 10^−6^ A

## Data Availability

The original contributions presented in this study are included in the article/Supplementary Materials. Further inquiries can be directed to the corresponding authors.

## Supplementary Materials

The following supporting information can be downloaded at: https://www.mdpi.com/article/10.3390/ma19143143/s1, Figure S1: High-resolution Li 1s XPS spectra of undoped and Li-diffused HPGe samples at different diffusion temperatures; Figure S2: Ge 3d high-resolution XPS spectra of HPGe samples after In diffusion at different temperatures. (a) 600 °C, (b) 700 °C, (c) 800 °C, and (d) 900 °C; Figure S3: In 3d high-resolution XPS spectra of HPGe samples after In diffusion at different temperatures. (a) 600 °C, (b) 700 °C, (c) 800 °C, and (d) 900 °C; Figure S4: Synchrotron μ-XRF mapping of the HPGe sample after In diffusion at 600 °C. (a,c) Two-dimensional fluorescence intensity maps; (b,d) corresponding three-dimensional intensity distributions..
