# Supplementary material for: Fabrication of Li/In Double-Sided Diffusion Contacts in Planar High-Purity Germanium Detectors and Their Low-Temperature X-Ray Response
Source: Materials (Basel). 2026 Jul 22;19(14):3143. doi: 10.3390/ma19143143 (PMC13413591; doi:10.3390/ma19143143)
Supplement: Supplementary file 1 [file materials-19-03143-s001.zip › materials-4382440-supplementary.pdf]

Fabrication of Li/In Double-Sided Diffusion Contacts in Planar High-Purity Germanium Detectors and Their Low-Temperature X-Ray Response

Meng Cao[1,2,*],Zexin Wang[1,†], Yanggang Jia[1,†], Qingzhi Hu[1]， Zhaoran Guan[1], Haofei Huang[1,*], Linjun Wang[1,2,3,*], Jian Huang[1,2,3]

[1] State Key Laboratory of Materials for Advanced Nuclear Energy & School of Materials Science and Engineering, Shanghai University, Shanghai 200444, China

[2] Zhejiang Institute of Advanced Materials, Shanghai University, Jiashan, 314113, China

[3] Shanghai Engineering Research Center for Integrated Circuits and Advanced Display Materials, Shanghai University, Shanghai 200444, China

* To whom correspondence should be addressed:

Corresponding author: Meng Cao; Haofei Huang; Linjun Wang

*E-mail address: caomeng@shu.edu.cn;

kardson@shu.edu.cn;

ljwang@shu.edu.cn

[†]: Zexin Wang and Yanggang Jia contributed equally to this work.

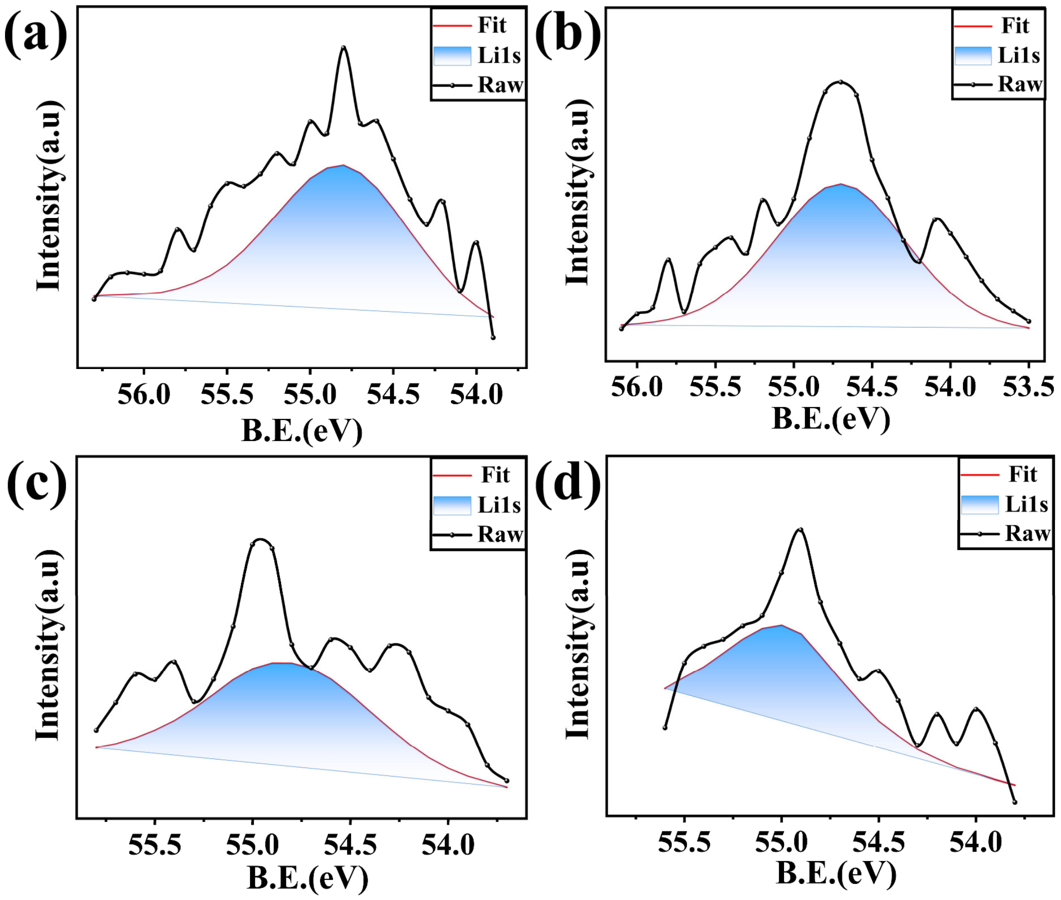

Figure S1. High-resolution Li 1s XPS spectra of undoped and Li-diffused HPGe samples at different diffusion temperatures.

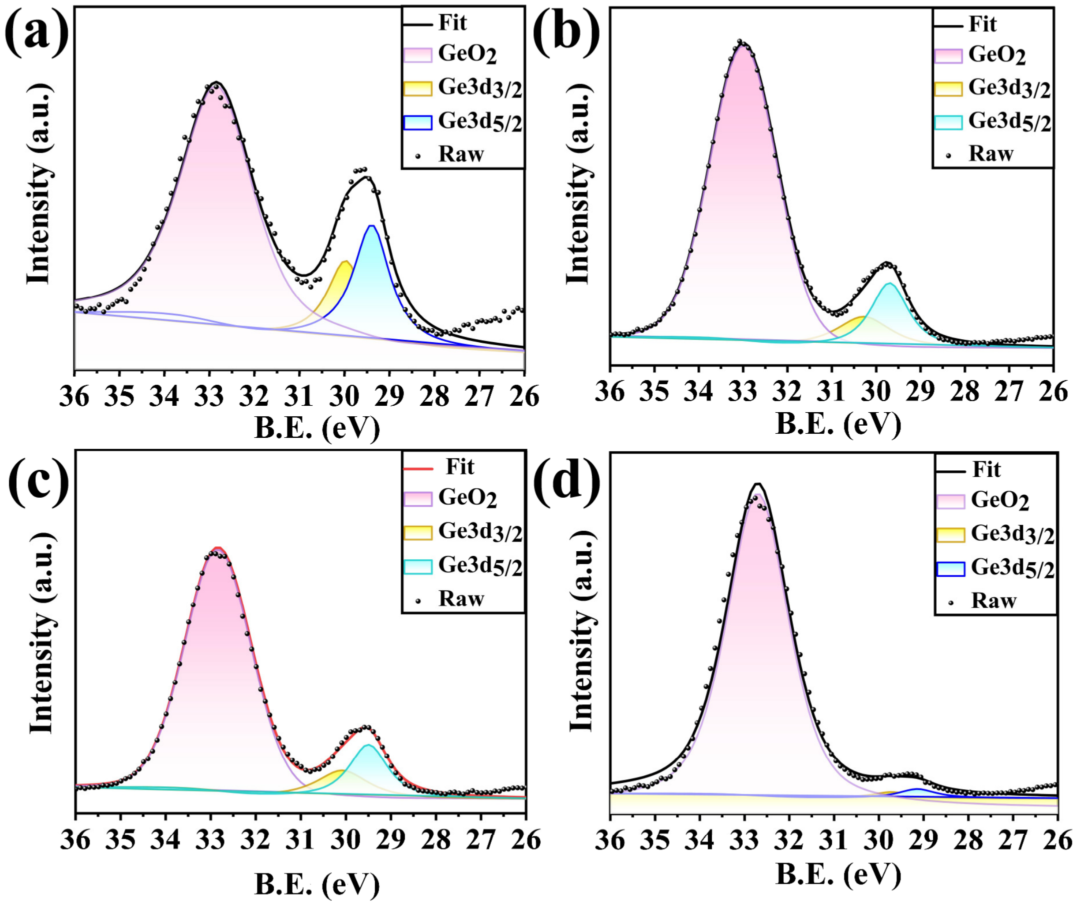

Figure S2. Ge 3d high-resolution XPS spectra of HPGe samples after In diffusion at different temperatures: (a) 600 °C, (b) 700 °C, (c) 800 °C, and (d) 900 °C.

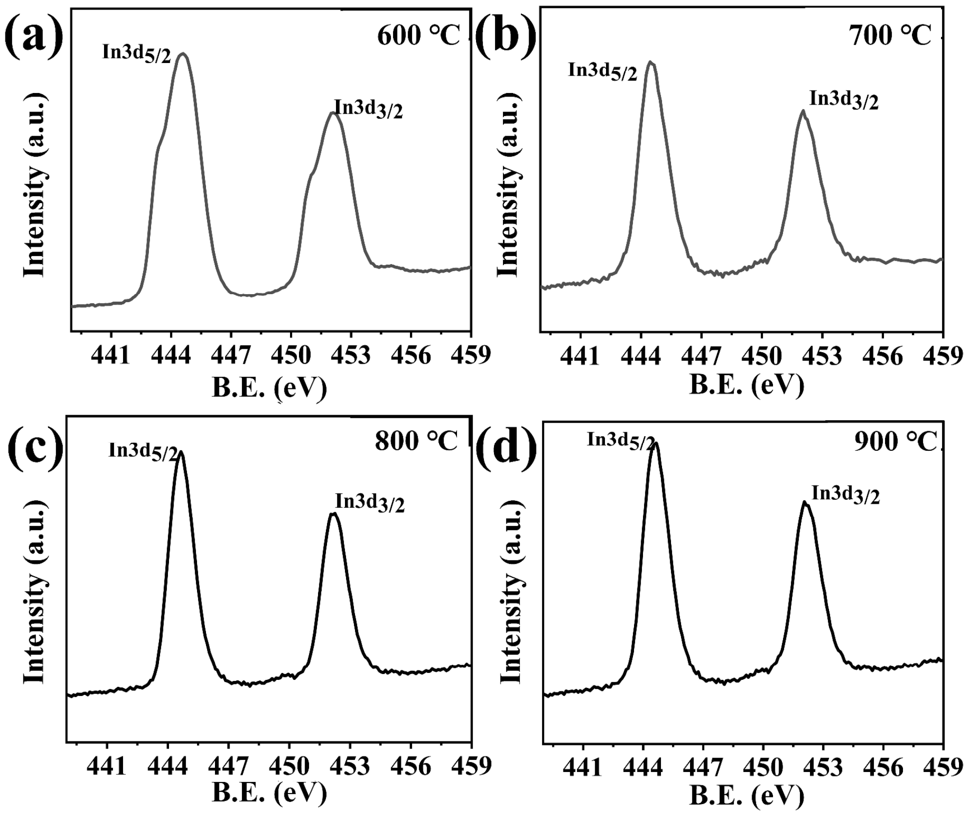

Figure S3. In 3d high-resolution XPS spectra of HPGe samples after In diffusion at different temperatures: (a) 600 ℃, (b) 700 ℃, (c) 800 ℃, and (d) 900 ℃.

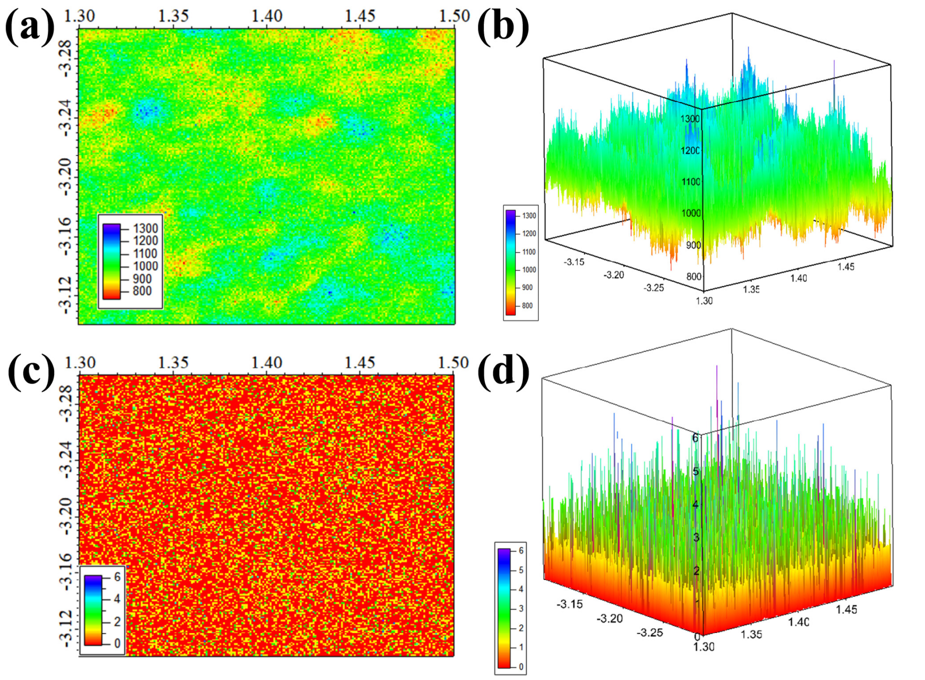

Figure S4. Synchrotron μ-XRF mapping of the HPGe sample after In diffusion at 600 °C. (a,c) Two-dimensional fluorescence intensity maps; (b,d) corresponding three-dimensional intensity distributions.
